# Phenotype of Transgenic Mice Carrying a Very Low Copy Number of the Mutant Human G93A Superoxide Dismutase-1 Gene Associated with Amyotrophic Lateral Sclerosis

**DOI:** 10.1371/journal.pone.0099879

**Published:** 2014-06-19

**Authors:** Jeffrey S. Deitch, Guillermo M. Alexander, Andrew Bensinger, Steven Yang, Juliann T. Jiang, Terry D. Heiman-Patterson

**Affiliations:** 1 Department of Neurology, Drexel University College of Medicine, Philadelphia, Pennsylvania, United States of America; 2 Philadelphia College of Osteopathic Medicine, Philadelphia, Pennsylvania, United States of America; University of Arkansas for Medical Sciences, United States of America

## Abstract

Amyotrophic lateral sclerosis (ALS) is a progressive neurodegenerative disease of the motor neuron. While most cases of ALS are sporadic, 10% are familial (FALS) with 20% of FALS caused by a mutation in the gene that codes for the enzyme Cu/Zn superoxide dismutase (SOD1). There is variability in sporadic ALS as well as FALS where even within the same family some siblings with the same mutation do not manifest disease. A transgenic (Tg) mouse model of FALS containing 25 copies of the mutant human SOD1 gene demonstrates motor neuron pathology and progressive weakness similar to ALS patients, leading to death at approximately 130 days. The onset of symptoms and survival of these transgenic mice are directly related to the number of copies of the mutant gene. We report the phenotype of a very low expressing (VLE) G93A SOD1 Tg carrying only 4 copies of the mutant G93ASOD1 gene. While weakness can start at 9 months, only 74% of mice 18 months or older demonstrate disease. The VLE mice show decreased motor neurons compared to wild-type mice as well as increased cytoplasmic translocation of TDP-43. In contrast to the standard G93A SOD1 Tg mouse which always develops motor weakness leading to death, not all VLE animals manifested clinical disease or shortened life span. In fact, approximately 20% of mice older than 24 months had no motor symptoms and only 18% of VLE mice older than 22 months reached end stage. Given the variable penetrance of clinical phenotype, prolonged survival, and protracted loss of motor neurons the VLE mouse provides a new tool that closely mimics human ALS. This tool will allow the study of pathologic events over time as well as the study of genetic and environmental modifiers that may not be causative, but can exacerbate or accelerate motor neuron disease.

## Introduction

Amyotrophic lateral sclerosis (ALS) is a degenerative disease affecting primarily the motor neurons in the spinal cord, brainstem, and motor cortex. Degeneration of the motor neurons leads to progressive paralysis, atrophy of denervated muscles, and ultimately death, with a median survival of less than 5 years. However, there is variability in ALS severity, with 20% of patients living longer than 5 years and 10% of patients living 10 years or more. Although most cases of ALS are sporadic (SALS), about 5–10% of ALS cases are familial (FALS), with approximately 20% of these resulting from mutations in the ubiquitously expressed Cu/Zn superoxide dismutase (SOD1) gene [1], [2]. Currently, 177 ALS causing mutations have been identified in the human SOD1 gene. There is significant heterogeneity of the disease reported in FALS subjects with SOD1 mutations [3]. Paradoxically, some siblings of FALS patients, possessing the same SOD1 mutation, do not show signs of the disease [4]. The role that SOD1 plays in the pathogenesis of ALS is not currently understood. The large variation in age of onset and severity in human ALS patients with specific SOD1 mutations lends support to the likelihood that there are other variables that determine disease expression. These variables likely include both environmental and genetic modifiers of disease [5], [6].

Transgenic (Tg) mouse models of FALS containing mutant human SOD1 genes have led to an explosion of research into the causes of ALS [7]–[9]. The most utilized and best characterized Tg mice are the G93A mutant hSOD1 [Tg(hSOD1-G93A)1GUR], abbreviated G93A, which develop motor neuron pathology and clinical symptoms remarkably similar to those seen in ALS patients [9]–[12]. These mice show onset with weakness of the hind limbs and tremors at approximately 90 days of age followed by hind limb paralysis and death by 120–150 days of age. The timing of symptoms is directly dependent on the number of copies of the transgene [11], [13].

The standard G93A mutant hSOD1 mouse model carries 25 copies of the mutant human SOD1 gene [14]. Loss of transgene copies can occur spontaneously during reproduction as a result of intra-locus recombination during meiosis. Our lab has developed a unique colony of G93A SOD1 mice that have only 4 copies of the G93ASOD1 gene with prolonged survival. These mice, designated as very low expressing (VLE) G93A SOD1 Tg, have a variable phenotype and do not always manifest clinical phenotype. Since the VLE mouse has a less penetrant phenotype with slower progression, it provides a model to study pathologic changes in sequence over a longer period of time so that the pathogenetic mechanisms can more easily be dissected. In addition, it also provides a model to study gene-environment interactions in disease expression as proposed in both familial and sporadic ALS [15]. The aim of this study is to characterize the clinical and pathologic phenotype of the VLE G93A SOD1 Tg mouse model of ALS which, like humans, has a variable penetrance.

## Materials and Methods

Mice used in this study originated from a colony of C57BI/6JXSJL/J Fn mice hemizygous for the human G93A SOD1 gene. The VLE mice were derived from a spontaneously generated founder male that possessed only 4 copies of the G93ASOD1 gene. Transgene copy number was verified for every animal using QPCR as previously described [5], [13].

**Figure 1:**
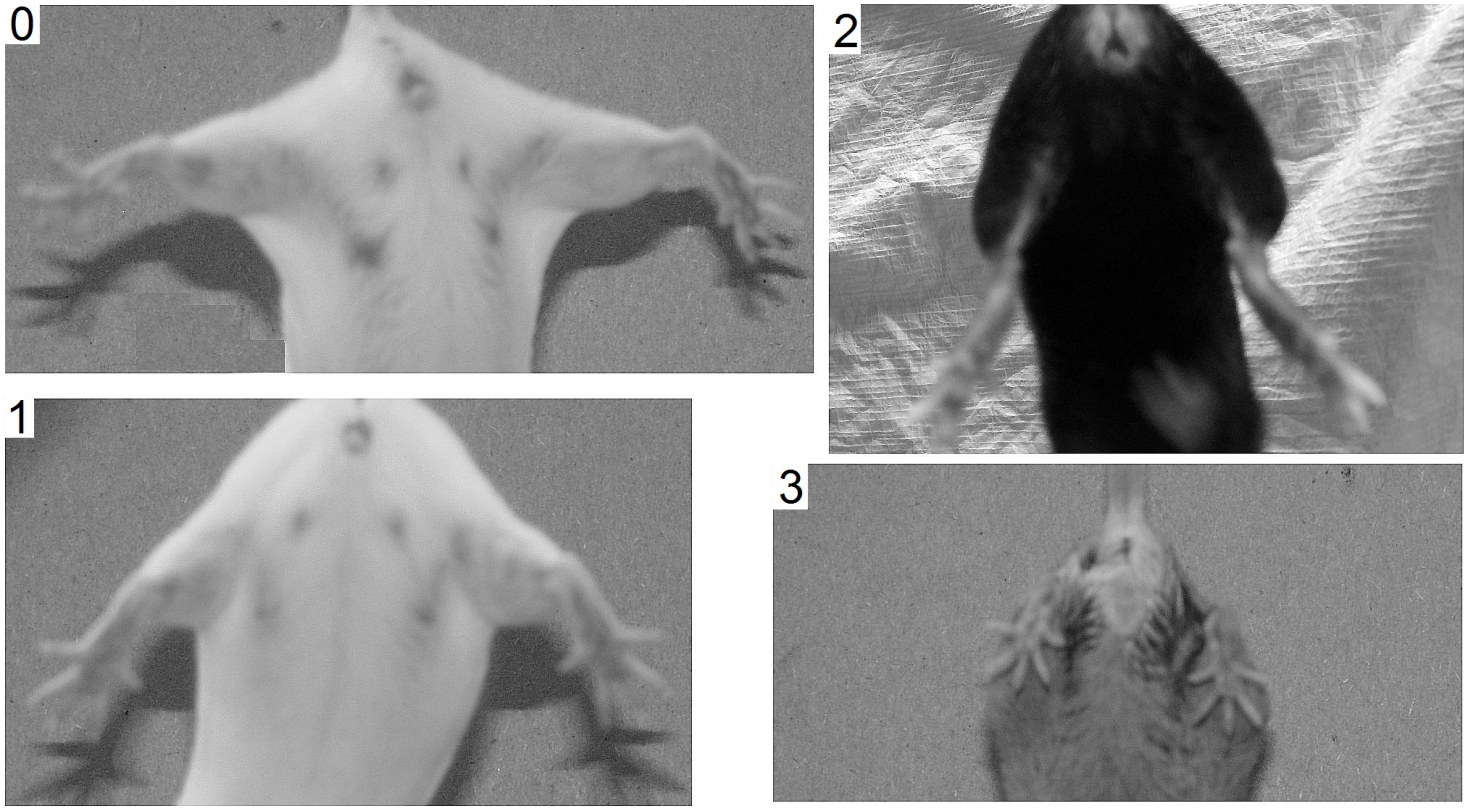
Splay reflex test. The splay reflex test, scored on a 0–3 scale, is performed by lifting the mouse by its tail and observing the degree of hind limb splay.

Two hundred and twenty three mice, 125 Tg+ VLE and 98 Tg-wild type controls, were evaluated for this study. Clinical status of each mouse was assessed by measuring weight and by ranking the splay reflex test on a 0–3 scale (Figure 1). Thirty three of these mice were used for lumbar spinal cord motor neuron counts and TDP-43 immunohistochemistry. The mice were sacrificed at three time points, 9, 18, and 24 months of age. If prior to the scheduled time point the mouse demonstrated paralysis of one hind limb, or was unable to right itself in 30 seconds when placed on its side, the animal was sacrificed. Natural death was not an endpoint in this study. Mice were euthanized by intraperitoneal injection of Beuthanasia-D (solution of sodium pentobarbital and sodium phenytoin, Schering, Kenilworth, NJ) at a dose containing 150 mg/kg pentobarbital followed by transcardial perfusion with 4% formaldehyde in 0.1 M phosphate buffered saline (PBS). The lumbar spinal cord was dissected out, embedded horizontally in paraffin, sectioned in 10 um-thick sections and mounted on gelatin-coated slides.

For neuron counts, every third section was stained with 1% Thionin. The ventral horns of the spinal cord were identified in relation to the presence of the central spinal canal, and all motor neurons in Rexed layer 9 of the lumbosacral segments were counted. All images were collected and analyzed with an Olympus BX60 microscope equipped with a DP70 digital camera and DP software (Olympus America, Center Valley, PA, USA). Motor neurons counts were performed by two independent counters and averaged to limit subjectivity.

The motor neurons of the spinal cord were also qualitatively examined for the presence of TDP-43. Immunohistochemistry was performed using an avidin–biotin–peroxidase complex system (Vectastain Elite ABC Peroxidase Kit; Vector Laboratories, Burlingame, CA). Briefly, paraffin was removed by xylene treatment, and the sections rehydrated through descending grades of alcohol up to water. Non-enzymatic antigen retrieval was performed by heating the sections to 95°C in 0.01 M sodium citrate buffer (pH 6.0) for 40 min in a vacuum oven. After a 30-min cooling period, the sections were rinsed, incubated in methanol/3% H_2_O_2_ for 20 min to quench endogenous peroxidase, and blocked with 0.1% bovine serum albumin (BSA) with 5% normal goat serum. Primary antibodies against TARDBP/TDP-43 (Proteintech, Chicago, IL; 1∶500) were added and the sections incubated over night at room temperature in a humidifier chamber. After rinsing, sections were incubated with biotinylated anti-rabbit secondary antibodies for 1 h, rinsed, and then incubated with avidin–biotin–peroxidase complex. Sections were developed with a diaminobenzidine (DAB) substrate and counter stained with Hematoxylin.

Motor neurons were characterized for the location of TDP-43 staining from 0 (pale cytoplasm with distinct nuclear staining) to 2 (translocation of the TDP-43 staining from the nucleus to the cytoplasm). The resulting scores were compared by age and between mutant and control mice and most importantly, the percent of neurons with translocation of TDP-43 to the cytoplasm was noted. TDP-43 for each animal was characterized in duplicate by two independent observers and averaged to limit subjectivity.

### Ethics

All animal procedures described in this study were carried out in accordance with and approved by the institutional animal care and use committee (IACUC) at Drexel University College of Medicine.

## Results

### Genotyping

All of the VLE mice were positive for human SOD1 by QPCR with a delta CT versus Interleukin-2 (IL-2) consistent with four copies of the transgene [13]. Transgene negative litter mates were used as controls.

### Clinical Status

**Figure 2:**
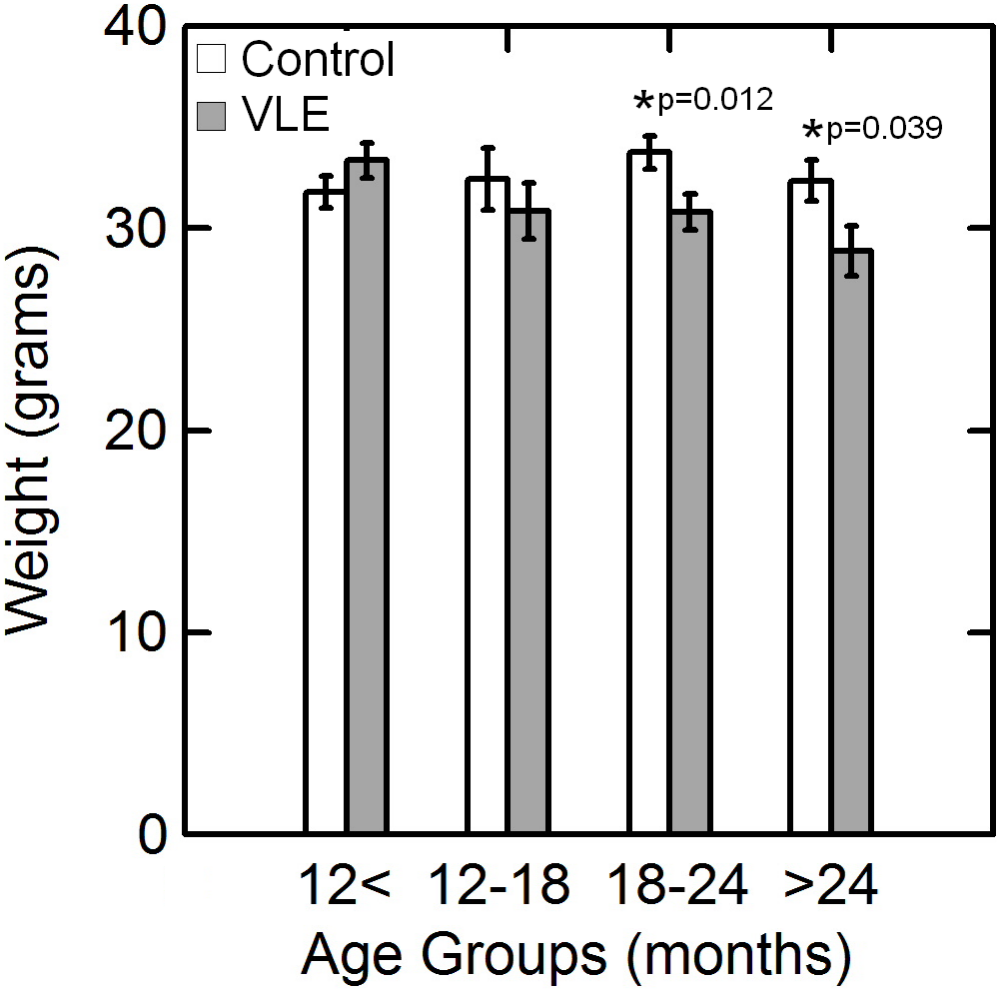
Variation in weight with age in male mice. Weight (mean ± standard error) of male VLE mice and control litter mates in four age groups. At ages less than 18 months old, there was no statistically significant difference (p>0.05) in the weight of male control mice as compared to the VLE animals. However, for ages greater than 18 months old, the male VLE mice demonstrated significant (p<0.05) weight loss as compared to their wild-type litter mates.

Significant weight loss can be used as an indicator of disease progression in high copy-number transgenic mice. There were significant (p<0.05) differences in weight at all ages between male and female mice (males heavier than females) in both transgene positive and negative mice, therefore weight loss as an indicator of motor neuron loss was evaluated separately by gender. Although female wild-type mice were on average slightly heavier than their transgenic litter mates, the difference between their weight at any of the ages examined did not reach statistical significance (p>0.05). For ages less than 18 months old, there was no statistically significant difference (p>0.05) in the weight of male transgenic mice as compared to their wild-type litter mates. However, for ages greater than 18 months old, the male transgene positive mice demonstrated significant (p<0.05) weight loss as compared to their wild-type litter mates (Figure 2).

**Figure 3:**
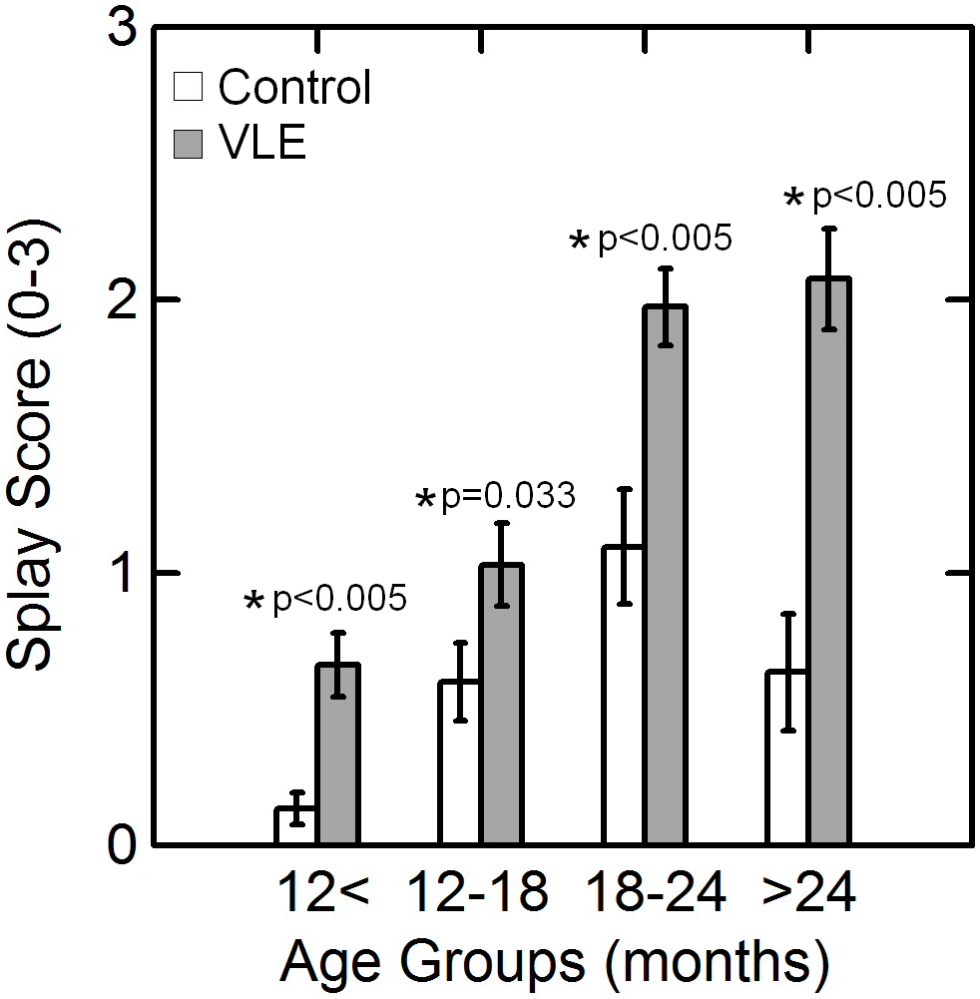
Variation in splay score with age. Splay score (mean ± standard error) of VLE mice and control litter mates in four age groups. The VLE mice demonstrated significant (p<0.05) greater splay scores as compared to their wild-type litter mates at all age groups.

There was significantly (p<0.05) increased motor weakness (evaluated with the splay reflex) in the VLE mice as compared to control animals (Figure 3). The transgenic mice demonstrated splay scores greater than 1 in 10% of the animals by 9 months. In mice between 9 and 18 months of age, 20% of animals showed splay >1 and 74% of mice older than 18 months showed splay >1. Hind limb paralysis was observed in VLE mice as early as 22 months. However, only 18% of VLE mice older than 22 months demonstrated hind limb paralysis. The average survival for animals that developed hind limb paralysis was 625.0±60.8 days. Not all VLE animals manifested clinically evident disease or shortened life span with approximately 20% of mice older than 24 months not manifesting any symptoms of motor neuron disease.

### Motor Neurons

**Figure 4:**
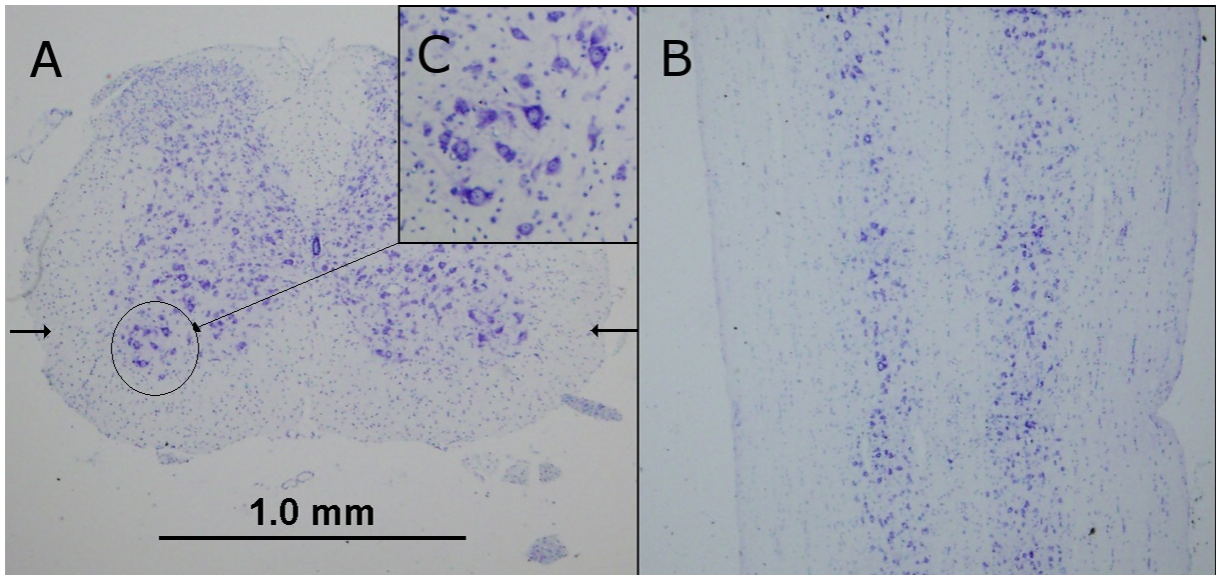
Lumbar spinal cord thionin stain. Lumbar mouse spinal cord 8 µm paraffin sections stained with thionin. [A] Transverse section. [B] Horizontal spinal cord section through the lumbar anterior horn at a level approximates by the arrows in [A]. [C] Motor neurons in Rexed layer 9.

**Figure 5:**
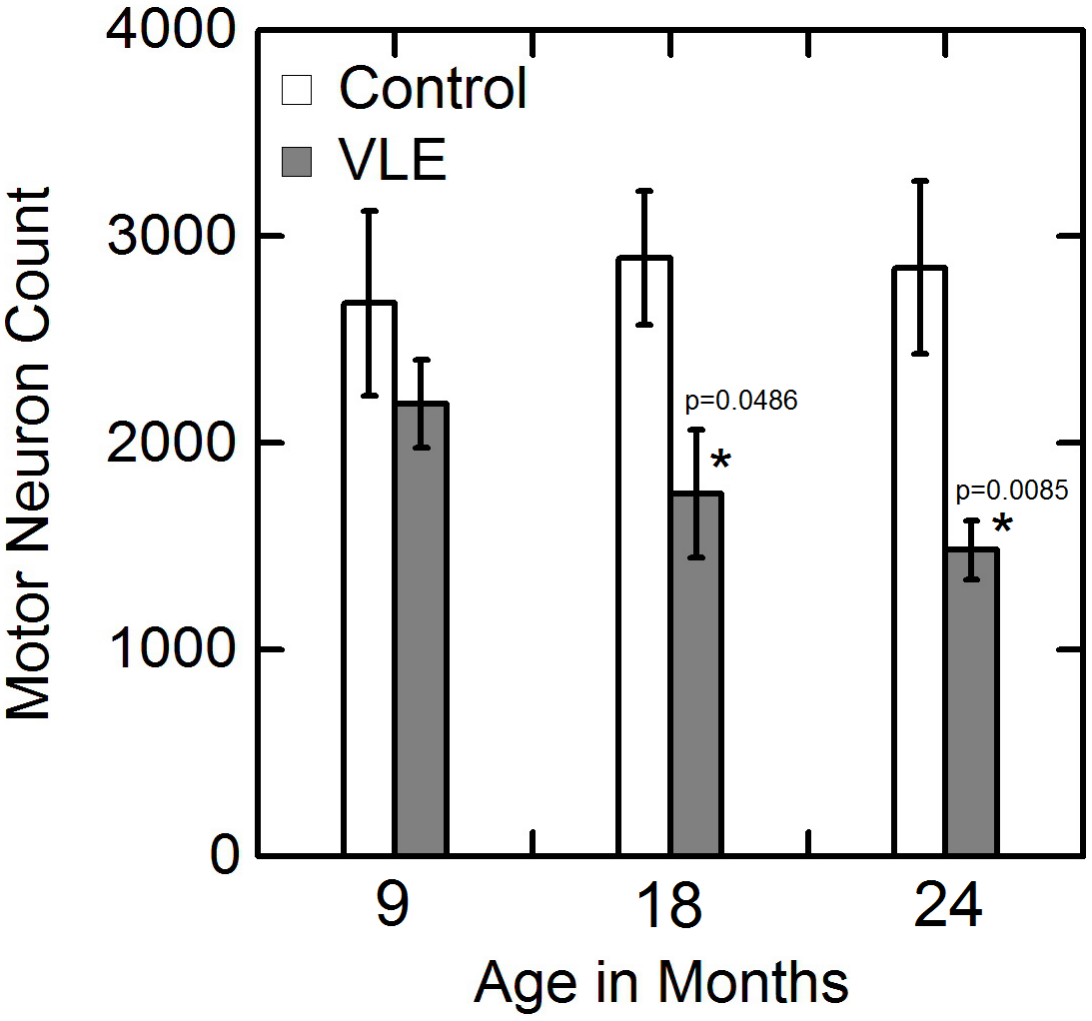
Variation in lumbosacral motor neuron numbers with age. Motor neuron numbers in the lumbosacral spinal cord a progressive reduction over time in the VLE mice as compared to the control animals. The decrease in motor neurons reached statistical significance in the 18 and 24 months old mice.

The alpha motor neurons, which are the largest cells (>30 µm diameter) in the spinal cord, are located in Rexed layer 9 and easily identified (Figure 4). The lumbosacral spinal cord alpha motor neurons counts for VLE and control mice are illustrated in Figure 5. The wild-type control mice demonstrated a stable number of motor neurons (2769±214, mean ± se) with no statistically significant difference in numbers (p>0.05) between the 9, 18 and 24 month old animals (p>0.05). In contrast, as compared to controls, the VLE mice showed a progressive decrease in the number of lumbosacral spinal cord motor neurons that reached statistical significance (p<0.05) in the 18 and 24 month old mice (Figure 5). Signs of individual degenerating motor neurons were observed in VLE mice as early as 9 month old with vacuolation even before significant neuronal loss.

### TDP-43 Immunohistochemistry

**Figure 6:**
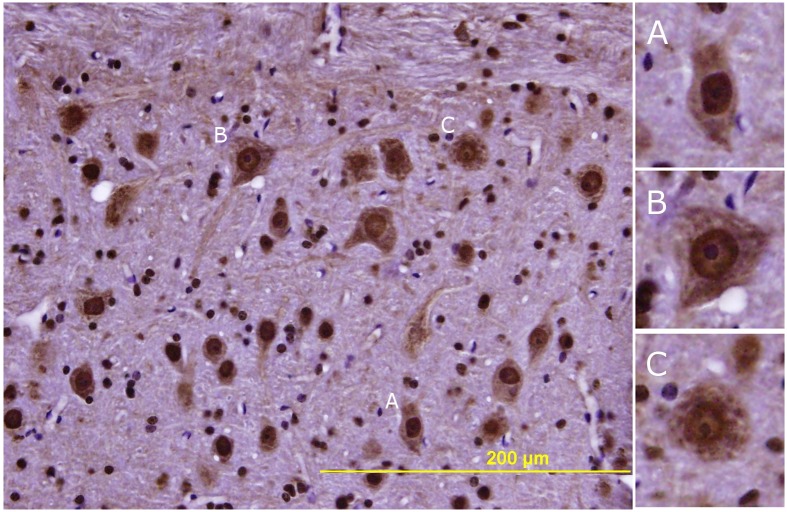
Lumbar spinal cord TDP-43 Immunohistochemistry. Characterization of TDP-43 staining in a 673 day old VLE mouse. [A] Motor neuron with normal TDP-43 distribution: a darkly stained nucleus and an even, lightly stained cytoplasm graded as a zero. [B] Demonstrates mixed localization with redistribution of TDP-43 into diffuse cytoplasmic staining with reduced label in the nucleus and is graded as a one. [C] Demonstrates motor neuron with redistribution of TDP-43 into punctate staining in the cytoplasm and nucleus and is scored as a two.

**Figure 7:**
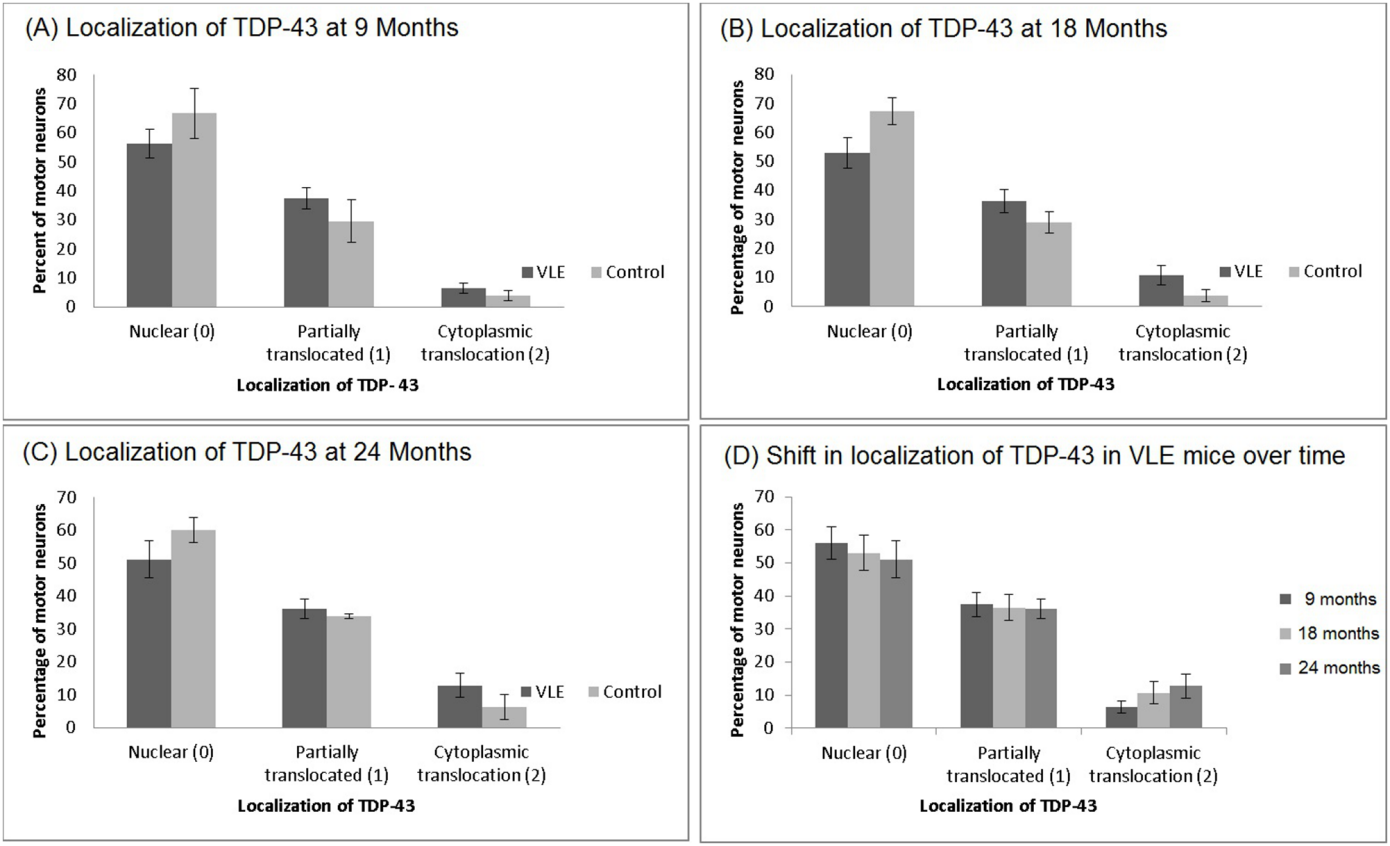
Translocation of TDP-43 with Age. Localization of TDP-43 in motor neurons of VLE and control mice sacrificed at 9, 18 and 24 months old (A, B, C). At each age there is an increase in the amount of motor neurons that demonstrate translocation of TDP-43 out of the nucleus into the cytoplasm in the VLE mice as compared to the control animals. These differences are statistically significant at each age at p<0.05. When only the VLE is shown over time (D) the increased percentage of motor neurons demonstrating translocation of TDP-43 is evident.

The distribution of TDP-43 immunoreactivity in spinal cord motor neurons is illustrated in Figure 6. In young animals, most TDP-43 is located in the nucleus with light cytoplasmic staining (Figure 6A). As the animals aged, the nuclear staining decreased and there was an increase in cytoplasmic staining (Figure 6B and 6C). This shift in TDP-43 immunoreactivity was more pronounced in the VLE mice as compared to their wild-type litter mates (Figure 7). In the 9 month old mice (Figure 7A) the percent of motor neurons in which the staining was nuclear only (score = 0) was 66.6±8.72% (mean ± sd) in control mice and 56.1±4.95% in VLE mice. There was partial translocation to the cytoplasm in 29.5±7.43% of the motor neurons in the control mice vs. 37.4±3.71% in VLE mice. TDP-43 was translocated to the cytoplasm in 3.82±1.69% of control motor neurons vs. 6.48±1.76% in VLE mice. This was a statistically significant difference (p = 0.0321).

In the 18 month old mice (Figure 7B), the percent of motor neurons with only nuclear staining (score = 0) in control mice was 67.2±4.62% compared to 53.0±5.33% in VLE. Partial translocation (score = 1) was observed in 28.9±3.66% of the motor neurons in the control mice compared to 36.5±3.98% in VLE mice. Cytoplasmic translocation (score = 2) was observed in 3.77±1.94% of the motor neurons in the control mice compared with 10.68±3.35% in VLE mice. These differences were all statistically significant (p<0.01).

In the 24 month old mice (Figure 7C), the percent of motor neurons with only nuclear staining (score = 0) in control mice was 60.0±4.62% compared to only 51.0±5.64% in VLE mice. There was partial translocation of TDP 43 to the cytoplasm in 33.7±0.77% of the motor neurons in the control mice compared to 36.1±2.98% in VLE mice. Cytoplasmic translocation (score = 2) was observed in 6.15±3.81% of the motor neurons in control mice compared to 12.84±3.61% in VLE mice. These differences were statistically significant (p<0.05) for nuclear localization and cytoplasmic translocation. In contrast to control mice, there was a consistent trend to cytoplasmic localization of TDP-43 in the VLE animals at each age and the cytoplasmic translocation of TDP-43 increased over time (Figure 7D).

## Discussion

We have demonstrated that VLE mice carrying 4 copies of the mutant human G93A SOD1 gene on the mixed B6/SJL background have clinical motor and pathologic changes with neuronal loss and translocation of TDP-43 when compared to controls yet, not all of these animals demonstrate weakness or die at an earlier age. While a previous report described a prolonged survival in a B6 G93A SOD1 mouse colony with reduced copy numbers, these mice all showed onset at 12 months with survival to 15 months [16], suggesting a copy number of less than 8 and greater than 4 based on our previously published work in B6SJL G93ASOD1 mice [13]. In contrast to the VLE mice, these mice did not demonstrate variable penetrance. The variable penetrance despite neuronal loss and translocation of TDP 43 support the VLE model as an important tool to dissect pathologic changes over time and to examine the interaction of environmental and genetic factors in ALS onset and progression.

VLE mice demonstrate motor neuron loss at a slower rate with a protracted time course as compared to the 25 copy G93ASOD1 animals. At 9 months of age, even before significant motor neuron loss, there was vacuolation of the motor neurons observed similar to 25 copy G93ASOD1 mice at 30 days of age. Two year old VLE mice lost approximately 50% of their motor neurons; a loss comparable to a 70 day old 25 copy G93ASOD1 mouse. By two years of age 80% of the VLE mice had clinically evident disease with about one fifth of these animals at end stage. The remaining animals did not manifest any clinical signs of disease. The average survival of mice demonstrating paralysis was 625 days compared with 129 days in the 25 copy mouse. Thus there is clearly a slower time course of neuronal loss and a clinically variable phenotype in the VLE mice as compared to the 25 copy G93ASOD1 animals. In contrast to other G93A SOD1 mutant mouse models which develop an ALS phenotype that eventually leads to hind limb paralysis and death, not all VLE animals manifested clinically evident disease or shortened life span. Only 18% of VLE mice older than 22 months demonstrated hind limb paralysis with approximately 20% of mice older than 24 months not manifesting any symptoms of motor neuron disease.

This slow progression of disease is likely to be more reflective of human SOD1 mediated ALS and will allow the sequential study of pathologic changes much more easily than the rapid course of the 25 copy mouse. In fact, it has previously been noted that translocation of TDP-43 was not a prominent feature of the G93A SOD1 transgenic mouse model carrying 25 copies of the transgene. However, the slow progression and prolonged survival of the VLE mouse allowed us to observe significant translocation of TDP-43 into the cytoplasm from the nucleus. This would further support the VLE mouse as a more realistic model of human disease than the 25 copy G93ASOD1 mouse.

Finally, similar to FALS kindred’s, not all VLE animals that carry the four copies of the transgene manifested clinically evident disease or shortened life span. In fact approximately 20% of these animals did not manifest any symptoms of motor neuron disease. This characteristic underscores the increased similarity of this model to human disease and will enable investigators to more easily study environmental and genetic factors that can provoke disease or alter phenotype by using this model. Simply, a comparison of the percentage of animals clinically affected, the timing of disease onset, and survival between groups of VLE mice who are exposed versus not exposed to a particular environmental factor will provide insight into the gene-environment interaction.

We recognize that variable phenotype could result from differential expression of mutant SOD1 by the VLE mice. However, we have previously reported on phenotype variability in transgenic mouse models of ALS [17]. Our study showed that in mice expressing the same number of G93A SOD1 transgene copies, the phenotype variability is mostly due to genetic background variability and not SOD1 expression. The authors feel that given the mixed genetic background (C57BL6/SJL) of the VLE mice most of the variability in ALS phenotype is likely due to genetic background variations and not differential expression of SOD1.

While we acknowledge that the slow progression and variable disease penetrance of the VLE model may demand longer time periods of study, we feel that using these animals in the appropriate way can actually accelerate our understanding of disease. In fact these animals would provide and ideal model to more easily study environmental and genetic factors that can provoke disease or alter phenotype. One example would be to examine the effects of pro-oxidant environmental risk factors and the genetic predisposition to the generation of free radicals in the VLE animals to determine the effects of exposure on the percentage of animals developing disease, the onset, the severity and the lifespan of affected animals. Furthermore, the slow tempo of neuronal loss and changes would enable a clearer picture of sequential events in the progression of motor neuron loss and the accompanying pathologic changes in spinal microglia and astrocytes.

In summary, we describe a new tool in the study of SOD1 mediated disease, the VLE mouse model with a variable penetrance of clinical phenotype, prolonged survival and protracted pathologic loss of motor neurons. These characteristics make the VLE mouse a valuable model for the study of genetic and environmental modifiers that may not be causative, but can exacerbate or accelerate motor neuron disease.
